# Western Journal

**Published:** 1844-01

**Authors:** 


### THE WESTERN JOURNAL.

NEW SERIES.—VOL. I.—NO. I.

LOUISVILLE, JANUARY 1, 1844.

### THE WESTERN JOURNAL.

It will be seen that the first number of our *New Series* makes its appearance enlarged in size, and printed on a new type. The publishers thus testify their disposition to render the Journal worthy of success, and although it must be admitted that they have not received as prompt payment from many of their subscribers as they had a right to expect, still they are determined to sustain the work. Arrangements have been made by which, in addition to the American journals now sent us in exchange, the most valuable of the foreign journals of medicine will be regularly received, and we shall have it in our power to give a summary, in each number, of the latest and most interesting medical intelligence. The publishers of medical books in our Atlantic cities have been in the habit of sending many of their valuable publications to the Journal, but hitherto they have not been regularly noticed. This has been a culpable omission, but the editors are not chiefly chargeable with it. The books, in most cases, were directed to gentlemen not connected with the Journal, under the mistaken belief that they would receive the notice due them. We intend, for the future, to bestow much more labor than herefore upon our department of Reviews and Bibliographical notices, and, with the understanding which now exists between the book-sellers and ourselves, we shall hereafter receive everything new relating to medicine which issues from the American press. If we are not greatly mistaken, we shall be able very decidedly to enhance the usefulness and interest of the Journal by this arrangement. Our readers may expect full analytical reviews of every new work recommended by the practical interest of its matter, and notices, however short, of all the publications that come to our hand. Most of the notices will necessarily be brief, but we shall endeavor to give such an account of each production as will enable the profession to judge of its claims to their favor.

Such are the improvements we have made in our plan of conducting the Journal. We are persuaded they will strike our readers as substantial, and we call upon them, once more, to second our efforts to produce a work worthy of the profession in the great Valley of the West. By a little exertion on the part of our friends, our subscription-list might be very greatly extended; thousands of physicians who are not now subscribers to any medical journal, might be induced to subscribe for it, and read it, to their own decided improvement, and the great benefit of their patients. May we not count on their efforts in behalf of our work, and may we not hope to receive more of the contributions of their pens? It is in their power to impart an interest to the pages of the Journal which cannot be given except by original communications. Histories of epidemics, or endemics, histories of individual cases of disease, or operations in surgery, all have their value, and impress a pleasing variety upon the pages of such a periodical. We know many scientific practitioners, many good writers, to whom we are sure this appeal will not be made in vain.

